# Distinct metabolic profile according to the shape of the oral glucose tolerance test curve is related to whole glucose excursion: a cross-sectional study

**DOI:** 10.1186/s12902-018-0286-7

**Published:** 2018-08-16

**Authors:** Leonardo de Andrade Mesquita, Luciana Pavan Antoniolli, Giordano Fabricio Cittolin-Santos, Fernando Gerchman

**Affiliations:** 1 Faculdade de Medicina da Universidade Federal do Rio Grande do Sul, Ramiro Barcelos, 2400, Porto Alegre, 90035-003 Brazil; 2 Serviço de Endocrinologia do Hospital de Clínicas de Porto Alegre, Ramiro Barcelos, 2350, Porto Alegre, 90035-903 Brazil

**Keywords:** Shape of the glucose curve, Area under the glucose curve, Metabolic syndrome, Insulin resistance, Diabetes mellitus

## Abstract

**Background:**

The shapes of the plasma glucose concentration curve during the oral glucose tolerance test are related to different metabolic risk profiles and future risk of type 2 DM. We sought to further analyze the relationship between the specific shapes and hyperglycemic states, the metabolic syndrome and hormones involved in carbohydrate and lipid metabolism, and to isolate the effect of the shape by adjusting for the area under the glucose curve.

**Methods:**

One hundred twenty one adult participants underwent a 2-h oral glucose tolerance test and were assigned to either the monophasic (*n* = 97) or the biphasic (*n* = 24) group based upon the rise and fall of their plasma glucose concentration. We evaluated anthropometric measures, blood pressure, lipid profile, high-sensitivity C-reactive protein, glycated hemoglobin, insulin sensitivity, beta-cell function, C-peptide, glucagon, adiponectin and pancreatic polypeptide.

**Results:**

Subjects with monophasic curves had higher fasting and 2-h plasma glucose levels, while presenting lower insulin sensitivity, beta-cell function, HDL cholesterol, adiponectin and pancreatic polypeptide levels. Prediabetes and metabolic syndrome had a higher prevalence in this group. Glycated hemoglobin, total cholesterol, triglycerides, high-sensitivity C-reactive protein and glucagon were not significantly different between groups. After adjusting for the area under the glucose curve, only the differences in the 1-h and 2-h plasma glucose concentrations and HDL cholesterol levels between the monophasic and biphasic groups remained statistically significant.

**Conclusions:**

Rates and intensity of metabolic dysfunction are higher in subjects with monophasic curves, who have lower insulin sensitivity and beta-cell function and a higher prevalence of prediabetes and metabolic syndrome. These differences, however, seem to be dependent on the area under the glucose curve.

**Electronic supplementary material:**

The online version of this article (10.1186/s12902-018-0286-7) contains supplementary material, which is available to authorized users.

## Background

Criteria for the diagnosis of diabetes mellitus (DM) and at-risk categories of glucose tolerance were established using the oral glucose tolerance test (OGTT) [1]. For this purpose, we currently use only plasma glucose measurements at fasting and 2 h after ingestion of 75 g of dextrose. Interestingly, insights into the natural history of glucose tolerance and DM have been derived from data such as the 1-h plasma glucose (1hPG) concentration [2–5], the relationship between the fasting and 2-h plasma glucose levels [6] and the shape of the glucose concentration curve.

Cross-sectional studies in diverse populations, including Latinos and obese youths [7–14], have assessed the shape of the glucose curve during the 2-h 75 g OGTT and demonstrated patterns associated with insulin resistance and beta-cell dysfunction. Two cohort studies [15, 16] showed a different future risk of impaired glucose metabolism and type 2 DM in individuals with distinct shapes of the OGTT glucose curve. Another cohort study [17] found a distinct risk of progression to type 1 DM according to the shape of the glucose curve in subjects with positivity for autoantibodies who were relatives of people with type 1 DM. On the other hand, a cross-sectional study [18] did not find different odds of prediabetes (PDM). Recent research in obese young subjects with distinct shapes of the glucose curve [19] demonstrated differences in free fatty acid response, plasma incretin levels and insulin sensitivity and insulin secretion, which were directly measured using the euglycemic hyperinsulinemic and hyperglycemic clamp techniques, respectively.

In the current study, we examined how the shape of the plasma glucose concentration curve during the OGTT relates to hyperglycemic states, the metabolic syndrome (MetS) and its components, and hormones involved in the carbohydrate and lipid metabolism. We also investigated whether any differences found were dependent only on the shape of the glucose curve.

## Methods

### Study design and setting

We performed a secondary analysis on data obtained between 2008 and 2015 from patients without a previous diagnosis of metabolic syndrome referred for outpatient care to the Metabolism Unit of Hospital de Clínicas de Porto Alegre, a tertiary hospital linked to Universidade Federal do Rio Grande do Sul, a public university in southern Brazil. These patients were enrolled in a cross-sectional study designed to examine the mechanisms and risk factors related to the development of type 2 diabetes and the metabolic syndrome. Additional information regarding the study protocol may be accessed elsewhere [20]. The study protocol was approved by the Institutional Review Board of Hospital de Clínicas de Porto Alegre.

### Subjects

We included in the analysis adult individuals who had a complete, 2-h OGTT with five equally spaced measurements of plasma glucose and insulin concentration. Exclusion criteria included insulin treatment, autoimmune diseases, uncompensated hypo or hyperthyroidism, malignant disease that could affect 5-year survival, stage IV-V chronic kidney disease, HIV infection, pregnancy or lactation, dementia, cirrhosis, hepatitis, glucocorticoid treatment and malnutrition. Application of the criteria above resulted in the exclusion of 41 subjects from an initial population of 228. Of the remaining 187 individuals, 31 were excluded from the analysis due to presenting glucose curve shapes that did not fit criteria for any group. We excluded 35 subjects with DM from the main analysis, because of the possible distortion of the results when including extremes of insulin resistance. The final sample size consisted of 121 individuals (156 for the alternative analysis including subjects with DM).

All subjects provided written informed consent.

### Measurements

We weighed subjects wearing light clothing without shoes. We used a stadiometer to measure height. We calculated body mass index (BMI) dividing the weight in kilograms by the height squared in meters. We measured waist circumference at the midpoint between the lower costal margin and the iliac crest, rounding values to the lowest 0.5 cm. We performed blood pressure (BP) measurements 1 week after the withdrawal of all antihypertensive medications. We measured office BP with an oscillometric monitor device (OMRON H-003D) with the appropriate cuff placed on the right arm of the patient, who had to be sitting for at least 5 min. We used the mean of the last two measurements to estimate systolic and diastolic BP.

Blood samples were taken after a 12-h overnight fast for analysis of plasma lipids (triglycerides, HDL and total cholesterol), glycated hemoglobin (HbA1c), high-sensitivity C-reactive protein (hs-CRP), adiponectin, glucagon, C-Peptide and pancreatic polypeptide (PP). Lipids were determined by an enzymatic method (Siemens ADVIA 1800 Chemistry System), HbA1c by high performance liquid chromatography (Tosoh Plus) and hs-CRP by turbidimetry (Siemens ADVIA 1800 Chemistry System). C-peptide was measured by chemiluminescent microparticle immunoassay (Abbott ARCHITECT; intra-assay coefficient of variation [CV] 2.7–3.2% and inter-assay CV < 10%). The enzyme-linked immunosorbent assay technique was used to determine glucagon (Yanaihara Institute; intra-assay CV < 5.1% and inter-assay CV < 18.9%), adiponectin (Invitrogen; intra-assay CV < 3.84% and inter-assay CV < 5.50%) and PP (Uscn Life Science; intra-assay CV < 10% and inter-assay CV < 12%).

After a 12-h overnight fast, subjects underwent a 75 g OGTT, with plasma glucose and serum insulin measured at baseline and 30, 60, 90 and 120 min. Plasma glucose was determined by an enzymatic method (Roche Cobas c501) and serum insulin by electrochemiluminescence (Centaur XP; inter-assay CV < 7.0%).

### Calculations

We estimated insulin sensitivity with data obtained from the OGTT, using the Gutt insulin sensitivity index [21]:

$$ {\displaystyle \begin{array}{l}\mathrm{Gutt}\ \mathrm{index}=\left\{\left[\left(75,000\ \mathrm{mg}+\left(\mathrm{FPG}-2\mathrm{hPG}\right)\times 0.19\times \mathrm{body}\ \mathrm{weight}\right)\div 120\ \min \right]\div \mathrm{mean}\ \mathrm{plasma}\ \mathrm{glucose}\right\}\div \log\ \\ {}\left(\mathrm{mean}\ \mathrm{serum}\ \mathrm{insulin}\right).\end{array}} $$

(FPG: fasting plasma glucose, 2hPG: 2-h plasma glucose; weight should be entered in kilograms, plasma glucose concentration in mg/dL and serum insulin levels in μU/mL)

This index was the most accurate in determining the presence of metabolic syndrome in our sample [22].

We calculated the insulinogenic index as the ratio between the changes in plasma insulin and glucose concentrations from baseline to 30 min after the oral glucose challenge, using the same units as in the Gutt index. We used the disposition index, obtained from the multiplication of the Gutt index and the insulinogenic index, to estimate beta-cell function.

### Classification of glucose curves

**Fig. 1:**
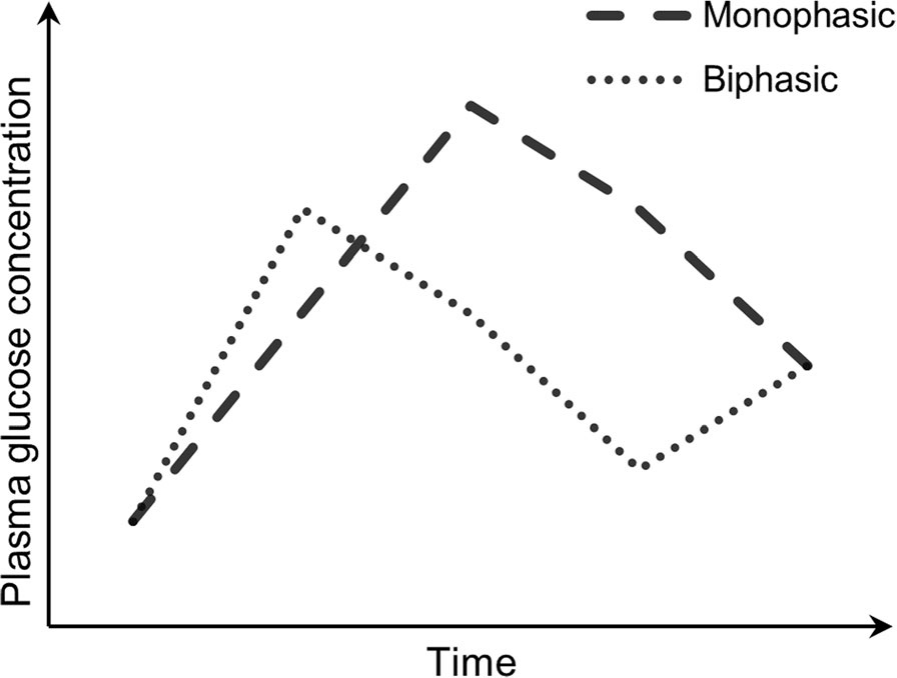
Shapes of the glucose curve: monophasic (lines) and biphasic (dots). M-shaped triphasic curves (not shown, n = 10) were included in the latter

We classified glucose curves according to previous studies [8, 12–14]. The “monophasic” (M) curve is defined by a rise in plasma glucose until a peak is reached, followed by a continuous fall. In subjects with a “biphasic” (B) curve, plasma glucose rises until a peak at 30′ or 60′, decreases and increases again from 90′ to 120′. In “triphasic” curves, plasma glucose increases from 0′ to 30′, decreases from 30′ to 60′, rises again from 60′ to 90′ and falls from 90′ to 120′. In this study, we included triphasic individuals (*n* = 10) in the biphasic group. We deemed a glucose curve shape “unclassifiable” if the difference in plasma glucose between 90′ and 120′ was lower than 0.25 mmol/L (except for triphasic curves, in which we applied this threshold to the change in plasma glucose between 60′ and 90′ instead). We also excluded subjects with a steady rise in plasma glucose concentration not followed by a fall, who did not fit into the previous groups. Models of each shape are shown in Fig. 1.

### Definition of glucose tolerance statuses and metabolic syndrome

We used the American Diabetes Association criteria (based on FPG and 2hPG) [1] not considering HbA1c to categorize subjects as having normal glucose tolerance (FPG < 5.6 mmol/L and 2hPG < 7.8 mmol/L), impaired fasting glucose (FPG 5.6–6.9 mmol/L, 2hPG < 7.8 mmol/L), impaired glucose tolerance (FPG < 5.6 mmol/L, 2hPG 7.8–11.0 mmol/L) or diabetes (FPG ≥ 7.0 mmol/L and/or 2hPG ≥ 11.1 mmol/L or use of medication for the control of DM). Subjects with either impaired fasting glucose or impaired glucose tolerance were considered to have prediabetes.

We defined the presence or absence of MetS according to the harmonization of metabolic syndrome criteria from the International Diabetes Federation, the American Heart Association and the National Heart, Lung and Blood Institute, among other organizations [23]. The chosen cut-off points for waist circumference were those of the previous International Diabetes Federation definition. We considered that a subject had MetS if he or she presented at least three of the following: waist circumference ≥ 94 cm for men or ≥ 80 cm for women; plasma triglyceride concentration ≥ 1.7 mmol/L or receiving drug treatment for this abnormality; HDL cholesterol < 1.0 mmol/L in males or < 1.3 mmol/L in females or receiving drug treatment for this abnormality; systolic BP ≥ 130 mmHg or diastolic BP ≥ 85 mmHg or receiving treatment for previously diagnosed hypertension; FPG ≥ 5.6 mmol/L or previous diagnosis of type 2 DM.

### Statistical analysis

All data are reported in SI units (except for HbA1c, the Gutt, insulinogenic and disposition indices, adiponectin, glucagon and pancreatic polypeptide) and expressed as absolute number (%), mean ± standard deviation, or median [P25-P75]. For continuous variables, we assessed the normality of distribution using the Kolgomorov-Smirnov and Shapiro-Wilk tests. We compared demographic characteristics and clinical and laboratory data between groups using the chi-squared test, the Student’s t-test or the Mann-Whitney U test as appropriate. We adjusted for confounding variables by means of ANCOVA for continuous dependent variables (after log or reciprocal transformation of variables with non-normal distributions) and multiple logistic regression analysis for categorical dependent variables. In these analyses, the shape of the glucose curve was included as categorical independent variable (or factor) and each suspected confounding variable as the covariate in separate models. A *p*-value < 0.05 was considered statistically significant. Statistical analyses were performed in PASW Statistics 18 (IBM Corporation, Armonk, NY, USA). We used G-Power 3.1 (Heinrich Heine Universität Düsseldorf, Düsseldorf, Germany) to conduct a *post-hoc* power analysis, in which we determined the β error probability of this study and the sample size required to achieve a power of 80% to detect, in the multiple logistic regression analysis adjusted for glucose AUC, a difference in the prevalence of the metabolic syndrome according to the shape of the glucose curve similar to the one we found in the unadjusted comparison, setting the alpha error rate to 0.05. We would need a total sample size of 231 subjects in order to reach such power in this analysis.

## Results

Data related to the OGTT and lipid profile were available for all subjects. For some subjects, data was missing as follows: waist circumference (1 female for B), blood pressure (2 for M, 1 for B), HbA1c (9 for M, 5 for B), hs-CRP (13 for M, 3 for B), adiponectin (13 for M, 3 for B), PP (28 for M, 10 for B), C-peptide (21 for M, 5 for B) and glucagon (39 for M, 13 for B).

97 subjects had glucose curves classified as monophasic and 24 as biphasic. The two groups had similar age, sex and ethnic composition. The monophasic group showed a trend toward obesity when compared to the biphasic group, with higher BMI and waist circumference (in both males and females), though these differences did not achieve statistical significance. Details on demographic and anthropometric characteristics are shown in Table 1.

**Table 1:** Demographic and clinical characteristics according to the shape of the plasma glucose curve

		Shape of the glucose curve		*p*-value
		Monophasic	Biphasic	
N		97	24	–
Age – years		51.96 ± 12.49	51.58 ± 10.73	0.893
Female sex – n (%)		73 (75.3)	20 (83.3)	0.401
White ethnicity – n (%)		82 (87.2)	18 (75.0)	0.137
BMI – kg/m^2^		31.36 ± 6.34	29.56 ± 4.48	0.192
Nutritional status – n (%)^a^	Lean	15 (15.5)	3 (12.5)	0.140
	Overweight	28 (28.9)	12 (50.0)	
	Obese	54 (55.7)	9 (37.5)	
Waist circumference – cm	Male	109.02 ± 14.98	94.00 ± 9.06	0.065
	Female	101.04 ± 14.42	96.71 ± 7.37	0.075
Blood pressure – mmHg	Systolic	136.93 ± 23.32	134.24 ± 18.93	0.608
	Diastolic	84.05 ± 13.00	86.63 ± 11.76	0.387

^a^Lean: BMI < 25 kg/m^2^; Overweight: BMI ≥ 25 kg/m^2^ and < 30 kg/m^2^; Obese: BMI ≥ 30 kg/m^2^

**Fig. 2:**
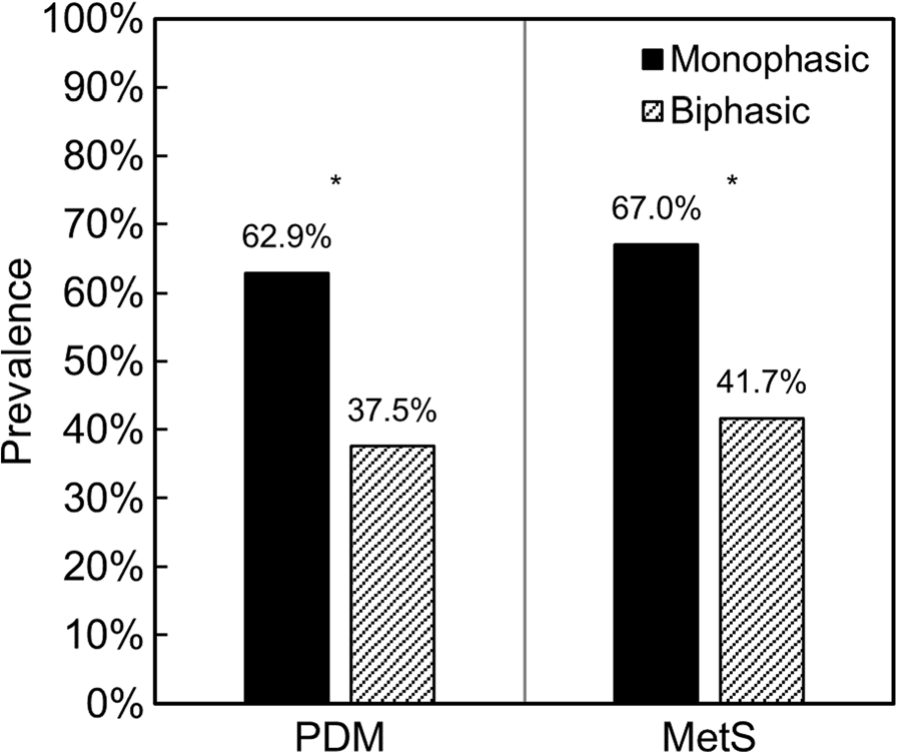
Prevalence of prediabetes and metabolic syndrome according to the shape of the glucose curve. * PDM: *p* = 0.024 (no adjustment), *p* = 0.120 (adjusted for waist circumference), *p* = 0.059 (adjusted for glucose AUC); MetS: *p* = 0.022 (no adjustment), *p* = 0.061 (adjusted for waist circumference), *p* = 0.772 (adjusted for glucose AUC)

The monophasic group had higher plasma glucose levels at all timepoints and a greater area under the glucose curve (glucose AUC). While fasting and 2-h serum insulin levels and plasma C-peptide concentration were also higher in this group, insulin sensitivity, disposition index, HDL cholesterol, adiponectin and PP levels were lower, and we found no statistically significant difference between groups in hs-CRP, total cholesterol, triglycerides or glucagon levels. The same group also presented a higher prevalence of prediabetes and metabolic syndrome. Data regarding laboratory characteristics are displayed in Table 2 and the prevalence of PDM and MetS is depicted in Fig. 2.

**Table 2:** Laboratory characteristics according to the shape of the glucose curve

	Shape of the glucose curve		*p*-value
	Monophasic	Biphasic	
N	97	24	–
FPG – mmol/L	5.38 ± 0.63	5.15 ± 0.44	0.044^ab^
30minPG – mmol/L	9.22 [8.19–10.53]	7.83 [6.76–9.04]	0.001^ab^
1hPG – mmol/L	9.83 [8.36–11.64]	6.03 [5.33–8.71]	< 0.001^ab^
90minPG – mmol/L	9.16 ± 2.40	6.60 ± 1.87	< 0.001^ab^
2hPG – mmol/L	8.11 [5.92–9.39]	6.33 [5.07–8.49]	0.028^ab^
Glucose AUC – mmol/L.h	17.55 ± 3.49	13.79 ± 2.99	< 0.001^a^
HbA1c - %	5.81 ± 0.63	5.74 ± 0.51	0.630
Fasting serum insulin – pmol/L	66.18 [45.15–100.47]	44.07 [32.10–67.65]	0.016^ab^
2-h serum insulin – pmol/L	476.70 [284.07–1017.51]	301.50 [148.98–470.16]	0.012^ab^
Gutt index	3.22 [2.43–4.12]	3.95 [3.06–5.29]	0.006^ab^
Insulinogenic index	0.91 [0.53–1.48]	1.48 [0.70–2.95]	0.017^ab^
Disposition index	2.99 [1.76–5.82]	6.42 [3.16–11.55]	0.002^ab^
C-Peptide – nmol/L	0.76 [0.43–0.96]	0.43 [0.38–0.70]	0.027^ab^
Glucagon – ng/L	332.50 [200.00–640.00]	600.00 [220.00–780.00]	0.115
Total cholesterol – mmol/L	5.41 ± 1.14	5.41 ± 1.10	0.990
HDL cholesterol – mmol/L	1.22 [1.01–1.37]	1.53 [1.23–1.76]	< 0.001^ab^
Triglycerides – mmol/L	1.54 [1.04–2.14]	1.29 [0.80–1.67]	0.106
hs-CRP – nmol/L	25.67 [11.90–67.83]	25.71 [13.48–63.29]	0.930
Adiponectin – μg/mL	11.93 [9.06–15.74]	15.42 [11.24–21.20]	0.018^ab^
PP – pg/mL	194.20 [103.65–392.15]	464.45 [204.90–808.15]	0.049^ab^

^a^After adjustment for waist circumference: 0.254 for FPG, 0.004 for 30minPG, < 0.001 for 1hPG, < 0.001 for 90minPG, 0.075 for 2hPG, < 0.001 for glucose AUC, 0.311 for fasting serum insulin, 0.050 for 2-h serum insulin, 0.024 for Gutt index, 0.011 for insulinogenic index, 0.012 for disposition index, 0.291 for C-peptide, 0.003 for HDL cholesterol, 0.094 for adiponectin and 0.096 for PP.

^b^After adjustment for glucose AUC: 0.273 for FPG, 0.583 for 30minPG, < 0.001 for 1hPG, 0.348 for 90minPG, 0.049 for 2hPG, 0.379 for fasting serum insulin, 0.436 for 2-h serum insulin, 0.767 for Gutt index, 0.464 for insulinogenic index, 0.819 for disposition index, 0.697 for C-peptide, 0.005 for HDL cholesterol, 0.420 for adiponectin and 0.205 for PP

In order to account for the potential confounding effect of the higher waist circumference in the monophasic group, we performed ANCOVA and logistic regression analyses. After adjustment, the differences in 30-min (30minPG), 1-h and 90-min (90minPG) plasma glucose concentration, glucose AUC, Gutt, insulinogenic and disposition indices, and HDL cholesterol levels remained statistically significant, and, while a trend for the monophasic group to have higher 2-h plasma glucose and serum insulin concentrations persisted, the differences did not reach statistical significance. Also, although not statistically significant, we observed a trend for the monophasic curve to predict the presence of the metabolic syndrome (odds ratio = 2.512 [95% CI: 0.959–6.585]).

We used the same approach to check whether the findings were related purely to the shape of the glucose curve or dependent on the whole glucose excursion, represented by the glucose AUC. In other words, we would like to know whether two subjects with the same glucose load, i.e. the same glucose AUC in the OGTT, but with different shapes of the glucose curve, would still present a diverse phenotype. Only the differences in 1hPG, 2hPG and HDL cholesterol levels achieved statistical significance after this adjustment. In the model, the monophasic curve showed a trend to have a negative effect on the probability of prediabetes (odds ratio = 0.186 [95% CI: 0.032–1.066]).

Considering data on the OGTT, clinical and laboratory characteristics were available for 35 subjects with DM, whom we did not include in the main analysis because of the possibility of distortion of the results due to their condition as a metabolic extreme, we performed an alternative analysis including those individuals (Additional file 1: Table S1). In this expanded sample of 128 monophasic and 28 biphasic individuals, the comparisons yielded mostly similar results to the main analysis. However, the difference in C-peptide levels between the groups did not achieve statistical significance, while the waist circumference (in males) was higher and glucagon levels were lower in the monophasic group before adjustment. Also, we observed statistically significant differences in PP and glucagon, after adjusting for waist circumference, and in waist circumference (in males), FPG, 90minPG, 2-h serum insulin, glucagon, PP and the prevalence of impaired glucose metabolism, after adjusting for glucose AUC. On the other hand, the Gutt index was not significantly different after adjustment for waist circumference and 2hPG was not significantly different after adjustment for glucose AUC.

## Discussion

This study demonstrates that subjects with monophasic glucose curves in the OGTT, compared with individuals with biphasic curves, have higher fasting plasma glucose and insulin concentrations at all time points. This group has, also, lower insulin sensitivity, insulin secretion, beta-cell function, HDL cholesterol, adiponectin and PP levels. The prevalence of impaired glucose metabolism and metabolic syndrome is also higher in the monophasic group. However, except for 1hPG, 2hPG and HDL cholesterol levels, these differences are not significant after adjusting for glucose AUC, a variable which reflects the whole glucose excursion these individuals are submitted to during the OGTT.

This relationship between the shape of the glucose curve and distinct metabolic profiles found in our study, with the monophasic group being at higher risk for metabolic dysfunction, corroborates the results of previous studies [8, 9, 11–14, 16, 18, 19]. Also, the finding that most differences were not significant after adjustment for glucose AUC is in agreement with the study by Tschritter et al. [8], which, to our knowledge, is the only one to have published results adjusted for glucose AUC, showing that most linear correlations between clinical/laboratory characteristics and the shape index (a quantitative measure for the shape of the glucose curve) were not significant. This added evidence brings up the hypothesis that the glucose AUC may be a better parameter for predicting metabolic dysfunction.

Surprisingly, even though the monophasic group has a higher prevalence of prediabetes in the unadjusted analysis, we found a trend for the monophasic curve to be associated with a lower probability of impaired glucose metabolism after adjustment for glucose AUC. We believe this may happen due to the diagnosis of prediabetes and diabetes being based on the fasting and 2-h plasma glucose values only [1]. Hence, if we consider subjects with the same glucose AUC, those who have an elevation at 120 min (biphasic) may be more prone to be diagnosed with impaired glucose metabolism. Nevertheless, since it did not reach statistical significance in the main analysis, this finding needs to be checked in high-powered studies.

In our study, in agreement with previous ones, subjects with biphasic curves displayed a better early-phase insulin secretion (as the insulinogenic index) [8, 9, 12–15]. This finding, along with the greater insulin sensitivity, may explain in part the biphasic curve, as plasma glucose concentration would fall in the early timepoints, followed by a rebound increase in the late OGTT [8, 9, 13, 14]. The exact reason for the existence of different shapes of the glucose curve, however, has not been elucidated yet. It may also involve differences in incretin levels and gastrointestinal physiology, requiring specific studies to explore the physiological intricacies behind the different shapes.

The main limitations of our study are the following. First, it has a limited sample size, with which we may not have been able to detect subtler differences between groups, especially in variables with a more significant proportion of missing data, such as the polypeptide hormones. HbA1c and hs-CRP also had a substantial amount of missing data, yet this had not affected the major outcome. As for the main analysis, we found that our study had a power of 53% and that we would need a total sample of 231 subjects to achieve a power of 80%. Hence, there is a need for studies with a higher sample size to analyze whether the null hypothesis of no difference in most metabolic parameters after controlling for glucose AUC holds true, since we expected to find the same differences despite the adjustment. Second, the cross-sectional design restrains the evaluation of the risk of developing impaired glucose metabolism and metabolic syndrome. Third, we performed a single execution of the OGTT, which does not allow us to make inferences on reproducibility of the shape of the glucose curve in this sample, something other studies [16, 24] have pointed out as relatively poor, and even describing that the combination of shapes of glucose curve from two OGTTs defines groups with different clinical and laboratory characteristics. Also, we estimated insulin sensitivity and beta-cell function with the Gutt and oral disposition indices respectively, instead of directly measuring them through the euglycemic-hyperinsulinemic and hyperglycemic clamp techniques. Nevertheless, previous studies have validated these surrogate measures against the gold standard clamp studies and demonstrated their relationship with clinical outcomes such as the presence of metabolic syndrome and future risk of diabetes [21, 22, 25]. Lastly, our classification of the shapes of the glucose curve - merging the biphasic and triphasic patterns and excluding individuals with a continuously rising plasma glucose concentration - is not based on solid scientific evidence, but is akin to the classification used in previous studies on the subject [8, 13, 18, 19].

Our study does not evaluate the use of the shape of the glucose curve as a tool in clinical practice. Our search did not return any article about the applicability of this parameter in the clinical setting (e.g. as criteria for differential testing or treatment). While the low reproducibility of this parameter and the neutralization of its impact after adjustment for the glucose AUC may limit its usefulness, specific studies are needed to give a definitive answer.

## Conclusions

The monophasic curve is associated with a greater prevalence of prediabetes and metabolic syndrome, as well as with higher plasma glucose and insulin levels, and lower insulin sensitivity and beta-cell function, but most of the differences found between the groups are driven by the area under the glucose curve. Future studies are necessary to examine the reasons behind the existence of distinct behaviors of the glucose curve and why they are associated with different metabolic and clinical phenotypes.

## Additional file

Additional file 1: **Table S1.** Demographic, clinical and laboratory characteristics according to the shape of the glucose curve in the full sample (including subjects with DM). (DOCX 19 kb)

## Abbreviations

1hPG: 1-h plasma glucose
2hPG: 2-h plasma glucose
30minPG: 30-min plasma glucose
90minPG: 90-min plasma glucose
B: Biphasic
BMI: Body mass index
BP: Blood pressure
CV: Coefficient of variation
DM: Diabetes mellitus
FPG: Fasting plasma glucose
glucose AUC: Area under the glucose curve
HbA1c: Glycated hemoglobin
hs-CRP: High-sensitivity C-reactive protein
M: Monophasic
MetS: Metabolic syndrome
OGTT: Oral glucose tolerance test
PDM: Prediabetes mellitus
PP: Pancreatic polypeptide
