# Supplementary material for: Distinct metabolic profile according to the shape of the oral glucose tolerance test curve is related to whole glucose excursion: a cross-sectional study
Source: BMC Endocr Disord. 2018 Aug 16;18:56. doi: 10.1186/s12902-018-0286-7 (PMC6097323; doi:10.1186/s12902-018-0286-7)
Supplement: Supplementary file 1 — Table S1. Demographic, clinical and laboratory characteristics according to the shape of the glucose curve in the full sample (including subjects with DM). (DOCX 19 kb) [file 12902_2018_286_MOESM1_ESM.docx]

Supplementary appendix 1. Demographic, clinical and laboratory characteristics according to the shape of the glucose curve in the full sample (including subjects with DM)

|  | | Shape of the glucose curve | | p-value |
| --- | --- | --- | --- | --- |
|  |  | Monophasic | Biphasic |  |
| N | | 128 | 28 |  |
| Age – years | | 52.30 ± 12.40 | 51.71 ± 10.05 | 0.814 |
| Female sex – n (%) | | 90 (70.3) | 22 (78.6) | 0.379 |
| White ethnicity – n (%) | | 107 (87.0) | 22 (78.6) | 0.254 |
| BMI – kg/m² | | 31.76 ± 6.35 | 29.60 ± 5.23 | 0.095 |
| Nutritional status – n (%) | Lean | 16 (12.5) | 4 (14.3) | 0.100 |
|  | Overweight | 39 (30.5) | 14 (50.0) |  |
|  | Obese | 73 (57) | 10 (35.7) |  |
| Waist circumference – cm | Male | 108.96 ± 13.16 | 96.33 ± 8.50 | 0.029 |
|  | Female | 102.30 ± 14.69 | 96.69 ± 10.44 | 0.101 |
| Blood pressure – mmHg | Systolic | 138.29 ± 24.11 | 136.17 ± 20.98 | 0.672 |
|  | Diastolic | 84.46 ± 13.64 | 86.91 ± 11.29 | 0.385 |
| FPG – mmol/L | | 5.64 [5.00-6.11] | 5.11 [5.00-5.56] | 0.038^#§^ |
| 30minPG – mmol/L | | 9.53 [8.39-11.15] | 8.14 [6.94-10.08] | 0.002^#§^ |
| 1hPG – mmol/L | | 10.83 [8.79-12.99] | 6.44 [5.38-11.21] | < 0.001^#§^ |
| 90minPG – mmol/L | | 10.03 [7.67-13.04] | 6.72 [5.76-8.63] | < 0.001^#§^ |
| 2hPG – mmol/L | | 8.92 [6.56-10.99] | 6.58 [5.63-9.47] | 0.018^#§^ |
| Glucose AUC – mmol/L.h | | 18.64 [16.00-22.59] | 13.81 [11.81-17.74] | < 0.001^#^ |
| HbA1c - % | | 6.00 [5.43-6.40] | 5.90 [5.45-6.35] | 0.652 |
| Fasting serum insulin – pmol/L | | 68.97 [45.68-101.25] | 49.65 [35.67-74.06] | 0.027^#§^ |
| 2-hour serum insulin – pmol/L | | 502.02 [293.66-995.76] | 349.53 [174.74-687.74] | 0.026^#§^ |
| Gutt index | | 2.65 [2.17-3.80] | 3.61 [2.93-5.05] | 0.005^#§^ |
| Insulinogenic index | | 0.79 [0.40-1.32] | 1.23 [0.51-2.34] | 0.010^#§^ |
| Disposition index | | 2.23 [0.97-4.48] | 5.79 [1.53-10.71] | 0.001^#§^ |
| C-peptide – nmol/L | | 0.76 [0.47-0.96] | 0.53 [0.39-0.83] | 0.070 |
| Glucagon – ng/L | | 282.50 [200.00-610.00] | 560.00 [280.00-855.00] | 0.033^#§^ |
| Total cholesterol – mmol/L | | 5.13 [4.55-5.99] | 5.31 [4.54-5.82] | 0.888 |
| HDL cholesterol – mmol/L | | 1.19 [0.99-1.37] | 1.45 [1.23-1.76] | < 0.001^#§^ |
| Triglycerides – mmol/L | | 1.54 [1.10-2.24] | 1.33 [0.80-1.65] | 0.077 |
| hs-CRP – nmol/L | | 30.48 [13.19-68.24] | 23.81 [11.43-63.29] | 0.619 |
| Adiponectin - µg/mL | | 11.18 [8.16-14.53] | 15.20 [10.98-19.75] | 0.011^#§^ |
| PP – pg/mL | | 191.10 [93.90-422.15] | 439.55 [251.13-656.85] | 0.013^#§^ |
| Glucose tolerance status – n (%) | Normal | 36 (28.1) | 15 (53.6) | 0.033 |
|  | Prediabetes | 61 (47.7) | 9 (32.1) |  |
|  | DM | 31 (24.2) | 4 (14.3) |  |
| Metabolic syndrome – n (%) | | 93 (72.7) | 13 (46.4) | 0.007 |

^#^ After adjustment for waist circumference: 0.314 for FPG, 0.012 for 30minPG, < 0.001 for 1hPG, < 0.001 for 90minPG, 0.084 for 2hPG, < 0.001 for glucose AUC, 0.452 for fasting serum insulin, 0.134 for 2-hour serum insulin, 0.069 for Gutt index, 0.006 for insulinogenic index, 0.009 for disposition index, 0.039 for glucagon, 0.002 for HDL cholesterol, 0.068 for adiponectin, 0.028 for PP, 0.120 for glucose tolerance status and 0.051 for metabolic syndrome.

^§^ After adjustment for glucose AUC: 0.035 for waist circumference in males, 0.005 for FPG, 0.758 for 30minPG, < 0.001 for 1hPG, < 0.001 for 90minPG, 0.286 for 2hPG, 0.205 for fasting serum insulin, 0.038 for 2-hour serum insulin, 0.788 for Gutt index, 0.256 for insulinogenic index, 0.459 for disposition index, 0.043 for glucagon, 0.001 for HDL cholesterol, 0.184 for adiponectin, 0.024 for PP, 0.029 for glucose tolerance status and 0.526 for metabolic syndrome.
